# HIV testing preferences among long distance truck drivers in Kenya: a discrete choice experiment

**DOI:** 10.1080/09540121.2017.1367086

**Published:** 2017-08-29

**Authors:** Michael Strauss, Gavin George, Emma Lansdell, Joanne E. Mantell, Kaymarlin Govender, Matthew Romo, Jacob Odhiambo, Eva Mwai, Eston N. Nyaga, Elizabeth A. Kelvin

**Affiliations:** a Health Economics and HIV and AIDS Research Division (HEARD), University of KwaZulu-Natal, Durban, South Africa; b HIV Center for Clinical and Behavioral Studies, Department of Psychiatry, Division of Gender, Sexuality and Health, New York State Psychiatric Institute & Columbia University, New York, NY, USA; c Department of Epidemiology and Biostatistics, CUNY Graduate School of Public Health and Health Policy, City University of New York, New York, NY, USA; d North Star Alliance, Nairobi, Kenya

**Keywords:** Discrete choice experiment, HIV testing, truck drivers Kenya, self-testing

## Abstract

Providing HIV testing services to truck drivers in Africa is crucial but has proven challenging. The introduction of HIV self-testing promises to provide expanded service delivery options for clients, potentially increasing demand for services and expanding coverage – especially important for high-risk and difficult-to-reach populations. This study examines the preferences regarding HIV testing service delivery models, among long distance truck drivers to identify testing services that would appeal to this population. Using a discrete choice experiment, this study examines the drivers of choice regarding HIV counselling and testing among 305 truck drivers recruited from two roadside wellness clinics along major trucking routes in Kenya. Participants made trade-offs between characteristics of HIV testing service delivery models by making hypothetical choices in a series of paired HIV testing scenarios. Conditional logit models were used to identify the HIV testing characteristics driving the selection of preferred scenarios, as well as determine whether preferences interact with individual characteristics – especially HIV testing history. Participants preferred free, provider-administered HIV testing at a roadside clinic, using a finger-prick test, with in-person counselling, undertaken in the shortest possible time. The strongest driver of choice was the cost of the test. Those who had never tested previously preferred oral testing and telephonic counselling, while those who were not regular testers favoured clinic based – over self-testing. The results of this study indicate that for the majority of participants – most of whom had tested before – the existing services offered at roadside clinics were the preferred service delivery model. The introduction of oral self-testing increases the options available to truck drivers and may even improve testing uptake for some, especially among those who have never tested before. However, these findings suggest the impact on HIV testing uptake of introducing oral self-testing may be limited in this population.

## Background

Targeting interventions to meet the needs of high-risk groups is crucial to mitigating the impact of HIV and AIDS (Schwartländer et al., 2011). Mobile populations have been identified to be at high risk of HIV infection due to their propensity to engage in concurrent relationships and transactional sex (International Labor Organization, 2005; Lafort et al., 2010). Long distance truck drivers in sub-Saharan Africa are particularly prone to acquiring HIV, with previous studies documenting prevalence rates as high as 26% in the region (Azuonwu, Erhabor, & Frank-Peterside, 2011; Botão et al., 2016; Bwayo, Omari, et al., 1991; Delany-Moretlwe et al., 2014; Rakwar et al., 1999; Ramjee & Gouws, 2002; Regondi, George, & Pillay, 2013). Increased risk among truck drivers has been attributed to the engagement in sex with female sex workers (FSWs) stationed along truck stops on major transport routes (International Labor Organization, 2005). Multiple concurrent sexual relationships with other regular and non-regular partners are also common (Lurie et al., 2003; Progressio, 2013), often paired with low levels of condom use (Bwayo, Mutere, et al., 1991), further increasing HIV risk. Additionally, studies have shown that truck drivers have inadequate access to health services (Delany-Moretlwe et al., 2014; International Labor Organization, 2005; IRIN, 2013; Ojo et al., 2011) – a concern not only for their own health but also increasing risk of HIV transmission to their sexual partners.

Facilities providing health services to truck drivers and FSWs appear along many major trucking routes (Lafort et al., 2010; North Star Alliance, 2015). The North Star Alliance (NSA), which currently operates in 10 sub-Saharan African countries, has increased the availability of HIV testing and counselling (HTC) to these populations, with coverage reaching 60% amongst clinic clients (Kelvin et al., 2017). However, not all truck drivers access clinics, and service delivery models must be optimised to ensure greater demand for HTC in line with national targets (UNAIDS, 2014), and especially in light of the ambitious UNAIDS Fast Track target that 90% of HIV positive people should know their status by 2020 (UNAIDS, 2015).

Advances in HIV testing technology have led to the introduction of self-testing as an additional option for clients, addressing potential barriers to HTC in clinical settings (WHO/UNITAID, 2016). A review examining public readiness for self-testing in Kenya found high acceptance among both providers and clients (Heard & Brown, 2016). The convenience, privacy and use of oral fluid associated with oral self-testing (rather than a blood test) were preferred by clients, while the main concern regarding self-testing was the potential lack of support following a positive test (Heard & Brown, 2016). However, it is unclear which characteristics are most important in driving HIV testing choices and whether preferences for certain forms of testing vary by population or past testing experience.

This study examines preferences regarding HIV testing modalities, including oral self-testing, among a sample of Kenyan truck drivers using a discrete choice experiment (DCE), in which participants were presented with hypothetical choices, making trade-offs between different characteristics of HTC services (de Bekker-Grob, Ryan, & Gerard, 2012; Lancsar & Louviere, 2008; Viney, Lancsar, & Louviere, 2002). More specifically, this study aims to identify the characteristics of service delivery models that drive choices when considering HTC, and determine which service delivery model truck drivers most prefer given their personal preferences and individual characteristics. Specifically, in the context of HTC, this study examines how service providers could adapt the way they provide HTC to truck drivers to reduce barriers and increase uptake.

## Methods

### Theoretical framework

Since the 1990s, DCEs have increasingly been used in healthcare to evaluate preference structures and help researchers and healthcare providers better understand demand for services (de Bekker-Grob et al., 2012; Ryan & Gerard, 2003; Weernink et al., 2014). Two economic theories underpin DCEs. First, Lancaster’s Theory of Consumer Choice (Louviere, Hensher, & Swait, 2000) states that consumers make choices that maximise utility based on the sum of the partwise utilities of characteristics (or attributes) of goods or services (Lancaster, 1966). This implies preferences regarding the attributes of a service drive choices rather than the service as a whole. In the context of HTC, this means preferences regarding service delivery model characteristics influence the decision to test. Given that a person will choose one service delivery model over another because they derive more utility from the combination of attributes that appear in that alternative than in the other, it is essential to understand the characteristics of HTC service delivery models that drive testing choices, to design HTC services that minimise barriers and maximise utility for clients.

Secondly, random utility theory states that utility derived from choice is comprised of a systematic component and a random component – this forms the basis for analysis of data (Thurstone, 1927). The systematic component comes from observable characteristics, both of the good or service and of the individual making the choice. The random component comprises any unobservable or unexplainable factors that contribute to overall utility, as well as measurement or specification error (Hensher, Rose, & Greene, 2005; Louviere et al., 2000).

### Study context and sample size

This DCE was situated within a randomised controlled trial (Kelvin et al., 2017), conducted at two of the eight NSA roadside clinics in Kenya, both located in Nakuru county, one of the highest HIV prevalence areas in the country (Kenya Ministry of Health, 2014; National STI and AIDS Control Programme (NASCOP), 2014). Participants were recruited from the waiting rooms in October through December 2015 and the eligibility criteria for inclusion in the study were: (1) at least 18 years old; (2) male; (3) employed as a truck driver; (4) primary residence in Kenya; (5) English or Kiswahili speaking; (6) HIV-negative or unknown HIV status (self-report); (7) able to sign the consent form (an ethics submission requirement); and (8) ability to receive payment for participation using MPesa (a cell phone-based money transfer system used in Kenya).

Participants were recruited by fieldworkers, who administered one-on-one baseline questionnaires containing demographic information, data on past HTC experiences, sexual history and risk behaviours, in English or Kiswahili. Participants were then randomised to either a choice arm (that offered participants oral self-testing or provider-administered finger-prick testing) or a control arm only offering provider-administered testing (Kelvin et al., 2017). The DCE was administered after the baseline survey, before randomisation.

305 participants, recruited in the RCT, were included in the DCE satisfying the minimum sample size of 125:

$$N\;\geq 500\frac{L}{SJ}$$

where *L* is the maximum number of levels for any attribute (four in our study), *S* is the number of choices in each choice set (two), and *J* the number of choices presented to each participant (eight) (Mele, 2008).

### DCE attributes

DCE choices were designed to elicit preferences regarding attributes of testing within the control of service providers (see Table 1). The levels for type of test, type of counselling and who administers the test were selected to capture key differences between standard testing at NSA roadside clinics and self-testing. Four location options were included to understand preferences relating to convenience and privacy. For time and cost, four options for each attribute were provided to allow variability for identifying patterns in preferences. Time options accounted for the entire HTC process, including waiting time. Cost was explained as an explicit fee for the HTC for consistency of interpretation; however, this is likely to be a proxy for the importance of money more broadly, including costs associated with accessing clinics.

**Table 1.** Attributes and levels used in the design the discrete choice experiment.

		Levels			
		Level 1 (Baseline*)	Level 2	Level 3	Level 4
Attributes	Type of test	Finger-prick blood test	Oral mouth-swab test	–	–
	Type of counselling	In-person counselling	Telephonic counselling	–	–
	Who administers the test	Nurse-administered	Self-administered	–	–
	Location	At a roadside clinic	At a clinic near home	At the company office	At home
	Time	90 min	20 min	40 min	3 h
	Cost	Free	You pay 250 Kenyan Shillings (approx. US$2.50)	You pay 300 Kenyan Shillings (approx. US$3.00)	We pay you 350 Kenyan Shillings (approx. US$3.50)

*Note: baseline characteristics shown in the Level 1 column are used as the reference category in the regression analysis.

A fractional factorial design was generated following Street, Burgess, and Louviere (2005, pp.463–467). We used a computer generated orthogonal main effects plan (OMEP) with 32 scenarios from the full factorial of 512 possible combinations of all the levels of the attributes. Along with the six attributes, a four-level blocking variable was included in the OMEP to efficiently divide the design equally into four questionnaire versions. Binary choice sets were constructed so that participants chose between two alternatives (“Option A” and “Option B”) in each of eight choice sets. The 32 scenarios generated in the OMEP were used as the first alternatives (Option A) in each of the 32 choice sets. These scenarios were generated to be orthogonal and ensure level balance for every attribute.

Option B for each choice set was generated by systematically adding 1 (cyclically) to each level of each attribute in Option A scenarios for every choice set to ensure an optimal design according to D-efficiency criteria (Street et al., 2005). This preserves both orthogonality and level balance, while ensuring zero overlap – in line with fundamental principles of efficient designs (Zwerina, Huber, & Kuhfeld, 1996). Participants were randomised to one of the four versions.

Choices had generically labelled alternatives – “Option A” and “Option B” – which provide no additional information about the service delivery model in either alternative. Questions were presented to participants on laminated cards, using words (English and Kiswahili) and pictures, by trained fieldworkers in one-on-one interviews using scripted instructions, containing detailed descriptions of the attributes and levels for consistency in the way participants understood tasks.

### Analysis strategy and model estimation

To estimate the main effects, dummy variables were created for each level of each attribute. The baseline scenario (reference group) was modelled on the typical HTC service available at a roadside clinic (see Table 1). To understand if preference structures interacted with participant characteristics (Strauss, George, & Rhodes, 2016), dummy variables (see Table 2) and interaction terms were created by multiplying dummy variables with attribute level dummy variables for inclusion in the model.

**Table 2.** Interaction variables used to stratify the analysis of the discrete choice experiment data including sexual behaviour and HIV testing history.

Individual characteristic	Dummy variable created from baseline questionnaire data
Ever tested for HIV	Ever tested = 1; never tested = 0.
Regular testing for HIV*	Regular tester = 1; non-regular tester = 0.
Multiple concurrent sex partners	Has one or more sex partner other than wife or main partner at home = 1; has no regular sex partner other than wife or main partner at home = 0.
Engages in sex with FSW	Paid for sex with money, gifts or a ride in the past six months = 1; did not engage in sex for money, gifts or a ride in the past 6 months = 0.
Consistent condom use	Always used a condom when having sex in the past 6 months = 1; sometimes or never used a condom when having sex in the past 6 months = 0.
Experience of testing at NSA roadside clinic	Ever tested at an NSA roadside clinic = 1; never tested at an NSA roadside clinic = 0.

*Note: In this paper we define regular testing as having tested more than once, and within the past 6 months in line with international guidelines for testing among high-risk populations (CDC, 2016; Mitchell & Horvath, 2013).

Conditional logit models were used to estimate parameters describing the strength, direction and statistical significance of associations between test attributes and the test chosen, and were estimated by:

$$Pr_{ij}=\frac{\text{exp}(\beta X_{ij})}{\sum_{k=1}^{K}\text{exp}(\beta X_{ik})},\text{ for all alternatives }K\;\text{in the choice set}$$

where *Pr_ij_* is the probability of individual *i* choosing alternative *j* in each binary set of alternatives *K*, β is a column vector of parameter estimates associated with $X_{ij}$, a row vector of the levels of the attributes in alternative *j* chosen by individual *i,* appropriate for analysis of data with a binary dependent variable (Haan, 2004). This model also allows estimates to be interpreted as odds ratios, allowing for easy comparison of preferences with the reference characteristic for each attribute.

A conditional logit model was selected in line with existing literature (Clark, Determann, Petrou, Moro, & de Bekker-Grob, 2014; de Bekker-Grob et al., 2012; Ryan & Bernard, 2003; Strauss et al., 2016) and in line with assumptions about the experimental design, preferred over a random effects logit model for binary outcomes. The main difference lies in assumptions made about the error terms. While both models assume errors are independent and identically distributed (IID), the random effects model relaxes the assumption of the independence of irrelevant alternatives (IIA) (Louviere et al., 2000). In this study design, both options were unlabelled so the presence of a third option in each choice set was unlikely to change the way participants viewed the first two options in relation to each other. Thus, violations of the IIA assumption were unlikely, and a conditional logit model was appropriate for parameter estimation. Further, results of a Hausman specification test returned a value of −23.23, interpreted as strong evidence that IIA holds (Hausman & McFadden, 1984). All analyses were conducted in Stata 13 using 95% determine significance.

#### Ethics

The study was approved by the Biomedical Research Ethics Committee at the University of KwaZulu-Natal in South Africa, the City University of New York Institutional Review Board and the Ethics Committees of the Kenya Medical Research Institute.

## Results

### Sample characteristics

Sample characteristics are shown in Table 3, including all variables used in the interaction analyses. The mean age was 37 years old (with a median of 37 years). The mean and median time spent away from home in the past month was 22 days. Only two participants had previous experience of self-testing. Participants were randomly assigned a version of the DCE, resulting in a fairly even distribution of participants across the different versions (27%; 31%; 21%; and 21% respectively).

**Table 3.** Key sample characteristics of 305 truck drivers recruited from 2 North Star Alliance clinics in Kenya, October to December 2015.

Individual Characteristic	*n*	%
*Age*		
20–29 years	58	19
30–39 years	145	48
Age 40–49 years	73	24
> 49 years	28	9
*HIV testing*		
Tested for HIV before	279	92
Regular tester	161	53
Ever tested at NSA roadside clinic	152	50
Came to clinic specifically for HIV testing	131	43
*Risk factors*		
Paid for sex with money, gifts or a ride in the past 6 months	179	59
Has one or more regular partners other than wife or main partner at home	142	47
Self-reported always using condoms (male or female) when having sex in the past 6 months	43	14

### Main effects

Table 4 shows the main effects estimates. Telephonic counselling (OR  = 0.881, *p* = 0.003); testing at the company office (OR = 0.826, *p* = 0.010); a three-hour test (OR = 0.776, *p* = 0.001); and any service fee (US$2.50 OR = 0.561, *p* < 0.001; US$3.00 OR = 0.351, *p* < 0.001) all decreased the odds of participants selecting a testing option, with the strongest effects for variables relating to cost. Reducing testing time to 20 min was the only characteristic that significantly increased the odds of testing (OR = 1.172, *p* = 0.034). Participants preferred not to test at home (OR = 0.864, *p* = 0.051), or to receive an incentive of US$3.50 (OR = 0.87, *p* = 0.056); however, these results were not significant. There were no significant differences in preferences between levels in other attributes, indicating an indifference within those attributes.

**Table 4.** Main effects conditional logit model (showing odds ratios), 305 truck drivers.

Attribute	Level (reference group shown in brackets)	OR	95% Confidence Interval		*p*-Value
Type of counselling	Telephonic counselling (in-person counselling)	0.881	0.809	0.959	0.003
Who administers the test	Self-testing (nurse-administered)	0.985	0.906	1.071	0.726
Type of test	Oral test (finger-prick blood test)	0.977	0.898	1.062	0.583
Location	Test at home (roadside clinic)	0.864	0.746	1.000	0.051
	Test at a clinic near home (roadside clinic)	0.907	0.767	1.073	0.257
	Test at work office (roadside clinic)	0.826	0.714	0.955	0.010
Time	20 min long test (90 min long test)	1.172	1.012	1.357	0.034
	40 min long test (90 min long test)	0.960	0.812	1.136	0.637
	3 h long test (90 min long test)	0.776	0.671	0.898	0.001
Cost	Test costs US$2.50 (no test cost)	0.561	0.485	0.650	0.000
	Test costs US$3.00 (no test cost)	0.351	0.297	0.415	0.001
	Receive US$3.50 (no test cost)	0.870	0.755	1.004	0.056
Number of observations	4828				
Log likelihood	−1558.694				
Pseudo R squared	0.068				
LR Chi squared (12)	229.1				

### Stratified analyses

**Figure 1.**
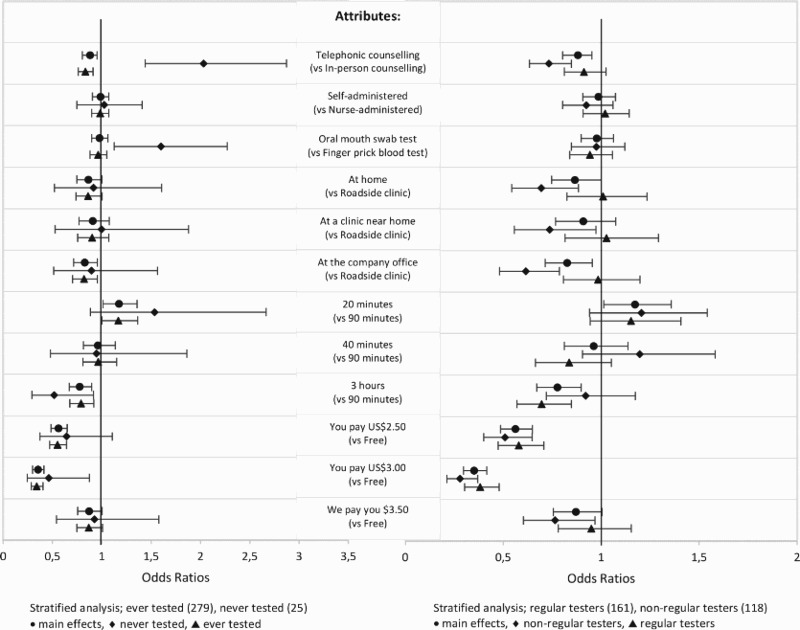
Conditional logit models stratified by HIV testing history. Point estimates are presented as odds ratios, and confidence intervals are estimated using 95% as a threshold for significance. When confidence intervals do not overlap, this indicates that there is a significant difference between the preferences across two groups.

Only two interaction models showed significantly different preferences between groups – having ever (versus never) tested for HIV, and of those that had tested, regular (versus non-regular) testing (CDC, 2016; Mitchell & Horvath, 2013). The left panel of Figure 1 shows aggregate and stratified results from participants who had ever tested. Participants that had never tested were significantly more likely to prefer oral testing over a finger-prick test (OR = 1.598, *p* = 0.009), while those that had tested were indifferent between an oral and finger-prick test. Those that had never tested were more likely to select telephonic counselling over in-person counselling (OR = 2.033, *p* < 0.001), while for those that had tested, telephonic counselling significantly decreased the odds of selecting a test (OR = 0.833, *p* < 0.001).

The right panel of Figure 1 shows results stratified by regular and non-regular testers. Regular testers were indifferent to service delivery characteristics for every attribute except time and money, with the odds of choosing a three-hour test significantly lower than a 90-minute test (OR = 0.695, *p* < 0.001) and a test with even a small cost significantly less preferable than a free test (US$2.50 OR = 0.579, *p* < 0.001; US$3.00 O = 0.381, *p* < 0.001). For non-regular testers, all significant preferences favoured the baseline test, with in-person counselling preferred over telephonic counselling (OR = 0.733, *p* < 0.001); testing at a roadside clinic preferred over testing at home (OR = 0.693, *p* = 0.003), a clinic near home (OR = 0.736, *p* = 0.031) or company office (OR = 0.615, *p* < 0.001) with a free test preferred over one they would have to pay for (US$2.50 OR = 0.509, *p* < 0.001; US$3.00 OR = 0.279, *p* < 0.001).

Interaction terms for risk factors other than HIV history, including condom use, number of regular sex partners and engagement with sex workers, were not significant. Thus, stratified models for these variables were not run. Analysis on participants having tested previously at a NSA roadside clinic revealed no significant differences in preferences with those who had tested elsewhere.

## Discussion

### Preferences for service delivery modalities

Given the high levels of HIV prevalence among truck drivers (Delany-Moretlwe et al., 2014), understanding how to best reach them with HIV testing services delivery models that will align with preferences and reduce barriers to testing needs to become a priority for reaching the ambitious targets of the UNAIDS Fast Track strategy (UNAIDS, 2015). Results from the main effects analysis of this study suggest that the baseline HTC service delivery model was best aligned with preferences for the participants in this study as a whole, with one exception: clients preferred shorter testing times. The baseline service delivery model closely mirrors that already provided at NSA roadside clinics – a free, nurse-administered, finger-prick test at a roadside clinic, where in-person counselling is provided by a healthcare worker, and where the entire process including queueing takes 90 min. This suggests that roadside clinics provide HTC in a way that is well aligned to the preferences of our study participants. This is somewhat unsurprising given that participants were recruited from the waiting rooms of NSA clinics. However, it is an indication that for these participants, rolling out self-testing is unlikely to significantly increase demand for HIV testing, and emphasises the importance of reaching clients that do not currently access NSA facilities with alternative HIV testing service delivery models. Increasing the number of well-staffed facilities along major trucking routes, and expanding the operating hours of existing facilities may help to reduce waiting times for clients, which is likely to improve the alignment of preferences with service delivery – although NSA clinics already offer expanded operating hours compared to other clinics (North Star Alliance, 2015).

Participants were indifferent between self and nurse-administered testing, as well as between oral and finger-prick testing. Based on these two characteristics, oral self-testing is not preferred by clients in general. Previous studies suggested that self-testing could increase convenience by allowing clients to test outside of the clinic environment (Heard & Brown, 2016). However, participants in this study preferred in-clinic testing to out-of-clinic testing, which is perhaps indicative of the value of support offered by healthcare workers in a clinical environment. Although clients were indifferent between roadside and community clinics, roadside clinics provide increased convenience – because they are located on the main trucking routes – and accessibility, given that participants spent on average 22 nights away from home in the past month.

Cost was by far the strongest driver of choice, with a fee of US$3.00 having the greatest negative effect on the odds of choosing a test. While testing is offered free in public health settings in Kenya, this finding has implications for the future roll-out of services, highlighting the importance of continuing to offer free testing and for reducing or eliminating costs associated with accessing testing services. If self-testing kits were available for purchase in pharmacies as some have suggested (Heard & Brown, 2016), this may have little effect on increasing testing uptake among this population, and given the mixed evidence for health outcomes and cost-effectiveness (Krause, Subklew-Sehume, Kenyon, & Colebunders, 2013; Linas, 2015; Maheswaran et al., 2016), should not replace in-clinic testing in the short term.

### Testing history and preferences

Preferences stratified by testing history, revealed no significant difference in preferences between nurse and self-administered testing, suggesting that while self-testing may be acceptable, it is not preferable, and unlikely to influence demand. However, participants who had never tested preferred oral testing over a finger-prick test. This may be due to a fear of needles, an often cited concern for clients (Heard & Brown, 2016; Strauss, Rhodes, & George, 2015). Offering oral testing (administered by a healthcare provider or the client) may result in increased testing uptake. Those who had never tested were indifferent regarding testing locations, but had a strong preference for telephonic counselling, perhaps indicating the importance of confidentiality, or the availability of additional support that could be accessed on demand.

For those who tested regularly, the drivers of choice were cost and, to a lesser extent, time. This suggests that the introduction of oral self-testing may not significantly alter testing practices among regular testers unless its introduction is associated with a reduction in time and cost. For non-regular testers, all significant drivers of choice favoured the baseline characteristics, especially with regards to the type of counselling (in-person), the location of the test (roadside clinic) and cost (free). There was no significant difference in preferences among the other attributes. Offering oral self-testing is unlikely to increase preferences and uptake amongst non-regular testers, however, there may be factors other than the service delivery model that hinder uptake.

### Limitations and future research

Participants in this study were recruited from clinic waiting rooms, with the majority having tested previously, and many already there to undertake HTC. Research is needed to understand which testing modalities align with preferences in the broader population, especially amongst truck drivers not accessing healthcare services. Information about HTC history and sexual risk behaviour was based on self-report, potentially leading to reporting errors as well as social desirability bias in how participants responded to questions about sensitive behaviours.

There was a small number of participants who had never tested (*n* = 25); therefore there may be other important drivers of choice undetectable due to the small sample size. Further research with larger samples is needed to identify weaker preferences. Finally, research in behavioural economics has shown that preferences are shaped by experience (Hoeffler & Ariely, 1999). Most of the participants in this study had no prior experience with self-testing, which may shape current preferences, and future research should examine how preferences change with experience. Further, self-testing provides the opportunity for expanding coverage to partners (both regular and casual), which could be important in this mobile population.

## Conclusion

The service delivery model for HTC used at NSA roadside clinics is well aligned with the stated preferences of this study sample. While the introduction of oral self-testing provides additional testing options for clients, it is unlikely to significantly alter HIV testing habits, especially amongst those that have tested before. Oral testing is more likely to align with the preferences of those who have never tested, but more so because it does not require a finger-prick than because it is a self-test, suggesting that introducing provider-administered oral testing in a clinical setting may result in similar outcomes. The stated preferences of the participants recruited at our NSA study sites suggest that the introduction of oral self-testing may have a limited immediate impact on their demand for HIV testing. Expanding this work to understand the preferences of truck drivers more broadly, especially those that do not currently access NSA clinics is vital for understanding the full effect that introducing oral self-testing is likely to have on demand for testing in order to help achieve the goals of the UNAIDS Fast Track targets (UNAIDS, 2015).
